# Review on Comparison of Different Energy Storage Technologies Used in Micro-Energy Harvesting, WSNs, Low-Cost Microelectronic Devices: Challenges and Recommendations

**DOI:** 10.3390/s21155041

**Published:** 2021-07-26

**Authors:** Amna Riaz, Mahidur R. Sarker, Mohamad Hanif Md Saad, Ramizi Mohamed

**Affiliations:** 1 Department of Electrical, Electronic and Systems Engineering, Faculty of Engineering and Built Environment, Universiti Kebangsaan Malaysia, Bangi 43600, Malaysia; p93122@siswa.ukm.edu.my (A.R.); ramizi@ukm.edu.my (R.M.); 2 Department of Electrical Engineering, Bahauddin Zakariya University, Punjab 60000, Pakistan; 3 Institute of IR 4.0, Universiti Kebangsaan Malaysia, Bangi 43600, Malaysia; hanifsaad@ukm.edu.my; 4 Industrial Engineering and Automotive, Campus de la Dehesa de la Villa, Nebrija University, Calle Pirineos, 55, 28040 Madrid, Spain

**Keywords:** energy storage system, low-cost microelectronics, WSNs, micro-energy harvesting

## Abstract

This paper reviews energy storage systems, in general, and for specific applications in low-cost micro-energy harvesting (MEH) systems, low-cost microelectronic devices, and wireless sensor networks (WSNs). With the development of electronic gadgets, low-cost microelectronic devices and WSNs, the need for an efficient, light and reliable energy storage device is increased. The current energy storage systems (ESS) have the disadvantages of self-discharging, energy density, life cycles, and cost. The ambient energy resources are the best option as an energy source, but the main challenge in harvesting energy from ambient sources is the instability of the source of energy. Due to the explosion of lithium batteries in many cases, and the pros associated with them, the design of an efficient device, which is more reliable and efficient than conventional batteries, is important. This review paper focused on the issues of the reliability and performance of electrical ESS, and, especially, discussed the technical challenges and suggested solutions for ESS (batteries, supercapacitors, and for a hybrid combination of supercapacitors and batteries) in detail. Nowadays, the main market of batteries is WSNs, but in the last decade, the world’s attention has turned toward supercapacitors as a good alternative of batteries. The main advantages of supercapacitors are their light weight, volume, greater life cycle, turbo charging/discharging, high energy density and power density, low cost, easy maintenance, and no pollution. This study reviews supercapacitors as a better alternative of batteries in low-cost electronic devices, WSNs, and MEH systems.

## 1. Introduction

**Figure 1:**
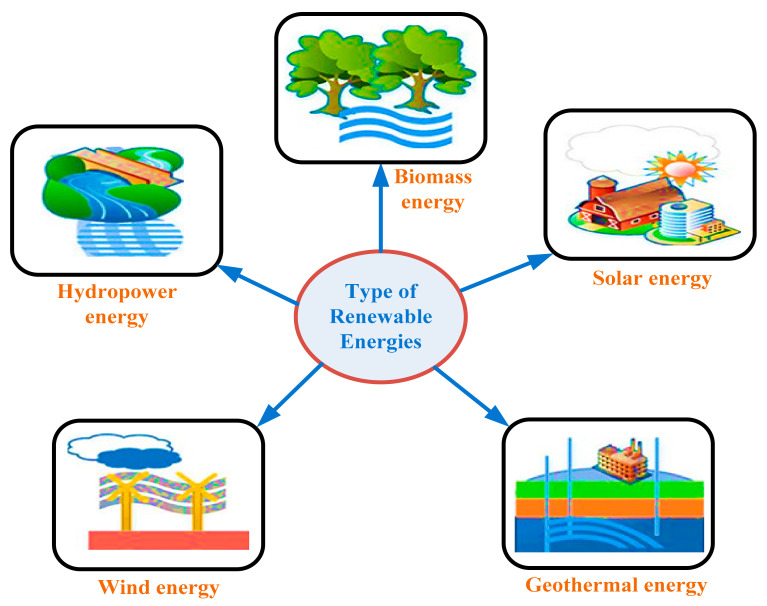
Renewable energy resources. Adapted from Ref. [10].

Electrical energy is so important in our life, in such a way that our lives totally depend on it. Electrical energy gives a faster life to everyone, i.e., fast manufacturing in industries, faster transport, and faster communication. All of these things have increased the demand for low-cost micro-electronic devices [1,2]. With the research developments, the conventional electronic devices are converted into portable electronic devices, wireless sensor networks (WSNs), and micro-energy harvesting (MEH) systems. WSNs gather information from a monitored field, with the help of low-cost sensors, and they communicate this information to distinct places, through wireless networks. Such applications include agricultural automation, medical implant, and different parameters (temperature, pressure, and acceleration) sensing in automobiles [3,4]. WSNs (network of sensors), low-cost microelectronic devices, and MEH systems are playing an important role in ambient intelligence [5]. The ambient intelligence system is an electronic system that is sensitive to the presence of humans. This WSNs technology is not only capable of gathering and sharing information from electronic machines globally, but also eliminates the human insolvent in difficult situations, and reduces the cost of communication. With the design of low-powered circuits, the energy required for these networks is also reduced considerably [6]. In spite of these developments, conventional batteries are still used as energy storage devices. In some situations, the replacement of these heavy batteries is very problematic [7]. Different methods have been developed for scavenging power from ambient energy sources, to make wireless networks self-sustained. In harvesting energy for such applications, ambient energy is a reliable and low-cost energy source. There are different forms of energy, such as solar, wind, biomass and thermal, etc. The ambient vibrational energy is a reliable energy source for many applications in our life [8,9]. In Figure 1, the following different renewable energy resources are shown: biomass, solar energy, and geothermal energy, hydro-power, and wind energies [10]. Our point of focus in this paper is only the power for microelectronic devices, energy for WSNs (nodes and sensors), and energy generated in micro-energy harvester’s, i.e., low-power and low-cost applications. These are the abundant forms of energy in our surroundings that are easily available and free of cost. 

**Figure 2:**
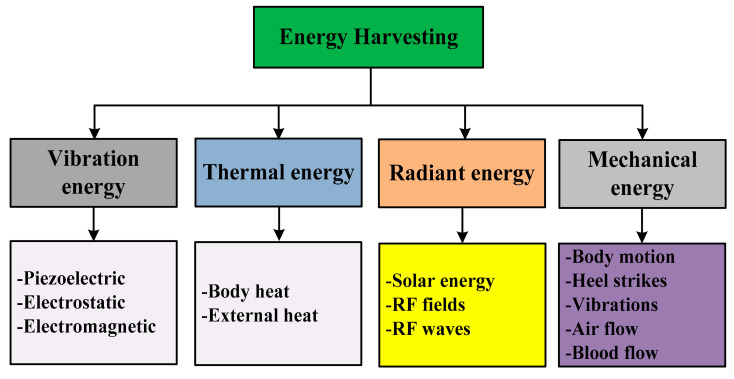
Ambiance energy harvesting.

In Figure 2, the different energy harvesting techniques to gain energy from the surrounding are shown. The thermal energy converts the heat energy from the sun to increase the temperature of a liquid (water), which converts into steam, and is then used to drive steam turbines to generate electrical energy [11]. 

Thermal energy is a source of renewable energy; we are interested in the energy on the micro-level, i.e., the thermal energy of sun waves, and heat energy of the human body, etc. [12]. It is one of the most environmentally friendly forms of energy. Radiant energy is a form of energy that travels by particles or waves; the most common examples are electromagnetic radiations, such as X-rays and heat emitted from the campfire, heat of the microwave, gamma rays, electricity, etc. [13]. In electromagnetic energy harvesting, the maximum power transfer to the load depends upon the inductance, and this inductance could be used to tune resonance to get the peak power [14]. However, in this energy harvesting, it is difficult to get the maximum power when nonlinearities are added into the system [15]. The main application of this technology is in telecommunication, radiometry, lighting, and heating. Radiant heat transmission is deadly [16]. The electromagnetic radiation of fire can kill or injure a passersby, or can cause the combustion material to catch on fire [17]. On the other hand, the kinetic and potential energies are the mechanical energy of an object. Few examples of kinetic mechanical energy are energy produced by light waves, the energy produced by electricity, and energy produced by sound waves [18,19,20]. The main disadvantage of mechanical energy is its safety issues and difficulty to transmit energy over long distances [21]. 

The power is generated by different techniques and resources, either by renewable or nonrenewable resources, but the main problem is the generation of low-cost electrical power and the design of an energy storage device that overcomes the problems that are associated with traditional batteries (self-discharging, loss of electrolyte, nickel gets stuck by hazards and burns faster, etc.). For the storage of the energy generated, there are only the following three options: batteries, fuel cells, and supercapacitors. Now supercapacitors are considered the best energy storage option. Supercapacitors have more capabilities than conventional capacitors and secondary ion batteries [22,23]. 

The need of low-cost electrical power turned the world’s attention towards renewable energy resources and low-cost MEH. The production of electrical energy by capturing free ambient energy from the surroundings, on a small scale, is known as MEH; a few examples are small wind turbines, the piezoelectric energy harvesting system, solar PV system, micro-hydro system, and hybrid systems [24]. Low-voltage electrical equipment in our daily life is very common and useful; this use voltages that are below 50 V. The low voltages are 48 V, 24 V, and 12 V. The different low-voltage devices in our homes are the doorbell, home security sensor, and garage door operator, etc. In micro-grids, the best choice for storage is supercapacitors, because of their temperature range, high energy density, and fast charging/discharging. One of the important low-cost MEH is piezoelectric energy harvesting, which converts vibrational energy into electrical energy [25,26,27]. 

## 2. Energy Storage Importance

For extra generated energy, there is a need for an efficient, reliable, and low-cost storage system [28]. This is one challenge, but delivering that energy to the consumers is another challenge. Storing and providing the extra energy to the consumers with less power loss, with a reduced cost, is very difficult [29]. Future technology in power engineering needs revolutionary changes. To reduce the cost and manage the energy demand, the world’s interest has turned towards the alternate sources of energy, i.e., renewable energy. The main challenge of the renewable energy systems is their dependence on some factors, which are difficult to control; one of these factors is the climatic conditions. The hybrid storage technology (combination of supercapacitor and battery) is very useful for storing and controlling power, and uses this power at a time of need [30]. The quality of a good energy storehouse is to provide power in the case of energy failures. Supercapacitors/batteries individually, or a merger of these, are the best examples of backup power devices, with lower equivalent series resistance (ESR). ESR is equivalent series resistance, for an ideal RC circuit, it represents the loss of useful energy in *R* and *C* as undesirable heat.

The recent developments in electrical energy storage (EES) showed good results with the hybrid combination of supercapacitors and lithium-ion batteries [31]. The hybrid EES system is good for low-voltage applications. In this hybrid EES system, the supercapacitor’s capacitance and power density is increased, and temperature problems can be neglected. For the design of a hybrid EES, great efforts have been made [32]; in these hybrid systems, supercapacitors and energy storage devices use different technologies, such as wireless power transfer (for power transmission) [33] or wind power [34], and low cost piezoelectric energy harvesting (as energy sources). In [35], the authors addressed the combination of supercapacitors and battery to design a hybrid EES with high-voltage efficiency. The availability of extra power depends upon the higher value of specific energy; lithium-ion batteries provide extra power than supercapacitors, on the basis of their higher specific energy.

The specific power of supercapacitors is more than lithium-ion batteries, which is the result of a low ESR. The recent study [36] has proposed that the life time could be increased by a hybrid combination of devices such as the battery, lithium-ion battery, and capacitor. One of the advantages of hybrid storage systems (lithium-ion batteries and supercapacitors) is the charging of the microelectronic devices by wireless power transfer [37]. Hybrid technology has the features of a high power density, short charging time, and long life. The main disadvantage of this hybrid storage system is discontinuity, matching of power, in the case of abundant energy resources, e.g., wind energy, vibrational energy. In [38], the authors proposed a new method of charging using supercapacitors, because the charging of the nodes in WSNs is the main issue. The supercapacitor empowers the sensor node to operate for a lifetime of 20 years, without any maintenance [39]. In many cases, supercapacitors make the system more efficient; for example, a supercapacitor makes the running of an elevator smoother with a switched-mode power supply [40]. The main problem in electric vehicles is the loss of kinetic energy, according to [41], the solution of this problem is regenerative braking by the application of supercapacitors; this also neglects the reduced efficiency of the battery. The combination of lithium-ion batteries and supercapacitors is doing very well in different electric applications, such as the following: toys, drones, low-cost microelectronic devices, and MEH [42,43]. One disadvantage of this proposed system is that the ESR of the battery is lower than the supercapacitors, so for the synchronization of these two devices, a feedback system is required. Now, the interest has turned towards the reduction in the size of electric gadgets, and nano/pico supercapacitors are the best examples of systems on chip (SoC) [44,45]. Low-cost MEH uses low-cost harvesting chips [46,47]. The future of WSNs is self-sustainability, in terms of power [48].

## 3. Electrical Energy Storage Classification

**Figure 3:**
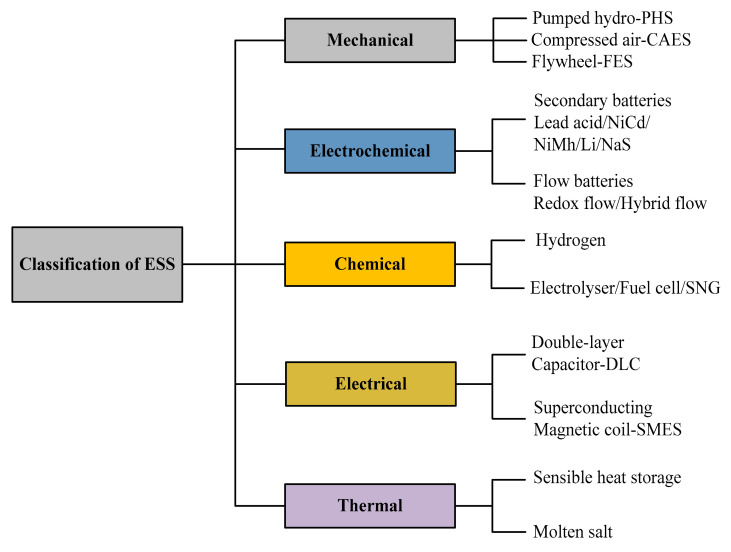
Classification of energy storage systems.

EES is divided into following types, according to their characteristics, as shown in Figure 3. Among the different storage technologies, we are considering only four, as described below.

### 3.1. Batteries

**Figure 4:**
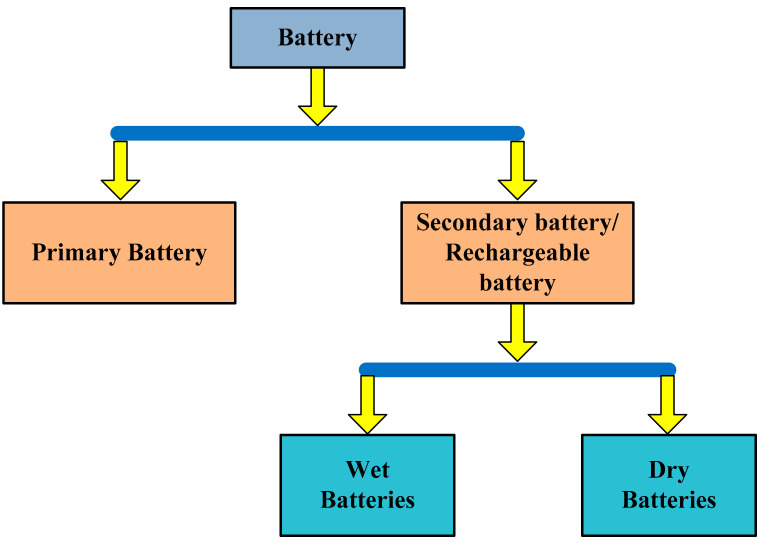
Different types of batteries.

Nowadays, batteries are commonly used in our daily life in most microelectronic and electrical devices; a few examples are cellular phones, clocks, laptops, computers, and toy cars [49,50,51]. Figure 4 shows the classification of various types of batteries. The electrical energy that is generated by different sources and techniques is stored in batteries, by chemical reaction. This energy can be converted back into electrical energy at any time. Conventional batteries are made up of two chemical metals, which form positive and negative electrodes. These are separated by a chemical medium called electrolyte. When an electrical circuit is connected between the battery terminals, due to an electrolytic chemical reaction, the positively charged ions start moving towards the cathode from the electrode, through the electrolyte. At the same time, the negatively charged ions move to the cathode, but the electrolyte creates a blockage and sends the current to the external electronic circuit [52,53]. The battery performance can be indicated by the following two indices: power density (maximum output power) and energy density (how much energy a battery stores). For example, in low-cost electrical devices, the energy storage capacity of the battery defines the operating timeline of that device. In modern batteries, nanomaterials are also used for the coating of the electrodes. When the battery is not in use, these nanomaterials separate the battery electrodes from liquid, which causes low discharging of the battery. Different types of nanomaterials are used, including carbon-coated silicon nanowires, carbon-nano tubes, etc. The main disadvantage of these materials is their low density, which causes high antiparticle resistance, and, as a result, the electrical conductivity of the battery is reduced. These materials also increase the cost of the batteries. 

**Figure 5:**
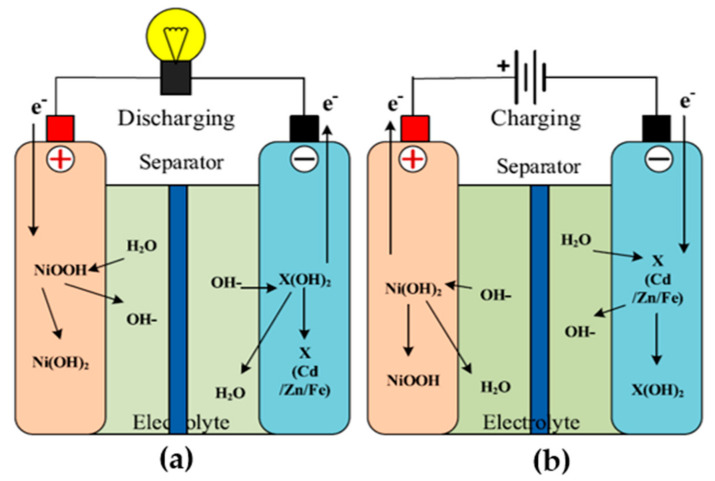
Chemistry of nickel-based battery, (**a**) during discharging, (**b**) during charging [57].

There are the following two types of batteries: primary and secondary. Primary batteries are for one-time application, as they have irreversible chemical reactions [54,55]. The price of these batteries is low and their application is simple for low-power devices. A few examples are as follows: zinc–carbon and zinc manganese alkaline batteries [56]. The specific applications are watches and torches. On the other hand, the application of secondary batteries is multiple times, as they are chargeable. Industries and automobiles are the main application of these batteries, where the value of the initial current should be high. However, the cost and the maintenance are the main disadvantages. The charging and discharging of the nickel battery is explained in detail, with the help of Figure 5. At the time of charge/discharge, the formation of Ni(OH)_2_, Fe/Cd/Zn(OH)_2_, and *M* occurs [57]. In the case of electric vehicles, (a) zinc and (b) nickel–iron batteries are not preferred, because of (a) their low specific power, (b) high cost, (c) low life cycle, and (d) maintenance requirement. On the other hand, the advantage of these two batteries is the 75% energy efficiency. 

### 3.2. Rechargeable Batteries

In this review, also considering rechargeable batteries as energy storage devices, the following are used by individuals: industry, commerce, microelectronic devices, and low-cost portable electronic devices [58,59]. The consumer batteries are mostly small and single-cell devices. On the other hand, the industrial batteries are large and multi-cell modules [60]. The battery (rechargeable) industry is growing day by day, due to an increase in the usage of portable devices, such as laptops, cell phones, notebook computers, etc. All these devices are battery powered. The demand for stationary batteries has also increased in industrial and individual sectors, to overcome the problem of electricity load shedding. Rechargeable batteries are available in different sizes, from a single cell used in toys and small devices to the bank of cells (large combination) that are used in many stand-by power systems, such as submarine propulsion and telephone exchanges. In the secondary batteries, the minimum life cycles depend upon the application. For example, the traction battery that is used in electric vehicles should have a minimum life cycle of 1000 cycles, whereas a solar rechargeable battery that is used in earth satellites have a life near 20,000 cycles. In Table 1, the different processes that affect the performance of different batteries are explained [61].

These deterioration processes cause battery failure due to different reasons, e.g., internal short circuit, progressive loss of capacity, and performance [62,63]. These deteriorative processes add up and cause the start of the decline in battery performance [64,65]. For the specific application of the battery, only life cycle is not the criterion of selection. The information that is necessary for managing battery systems is also important, e.g., energy storage capacity of the battery, how much run time is required before recharging, and the discharge rate [66]. Here, we will discuss some secondary batteries in detail, which are important in low-cost microelectronic devices, MEH, and WSNs. 

### 3.3. Lithium-Ion Batteries

According to [67], the lithium-ion batteries (LIBs) are the best source for a wide range of electric vehicles. The working principle of LIBs is the exchange of lithium ions between a cathode and an anode [68,69]. LIBs have higher specific energies than batteries made from other materials, such as zinc and lead, due to them being a low density and relatively lightweight [70]. Lithium is silver in color, and is a very soft metal. It is softer than lead, and has a body-centered cubic crystal structure. It is so light, with a specific density of approximately half of the water. Its density is 0.534 grams per cubic centimeter, and that of water is 1.000 grams per cubic centimeter. The melting point of lithium is 180.54 °C and boiling point is 1342 °C. 

The lithium-ion batteries also have more-stable overcharge/discharge cycles, because the radii of the lithium ion is small and causes fewer disruptions to the electrode structure during ion transfer. LIBs work by using the transfer of ions from the positive electrode to the negative electrode. At the positive electrode, *Li*^+^ is the result of the oxidation of lithium. These *Li*^+^ move to the negative electrode, where they become *LiCoO*_2_. As the lithium reactions are at both terminals, this battery can also be recharged by reverse operation. During discharge, the oxidation of lithium, from *Li* to *Li*^+^, through a reaction at the lithium–graphite-positive terminal is given below:

(1) $$Li_{x}C_{6}\Leftrightarrow xLi^{+}+xe^{-}+6C$$

In Equation (1), *Li_x_C*_6_ is a lithium–graphite compound. This reaction is also reversible and recharges the cell, leaving lithium cobalt oxide at the negative terminal, and it migrates back to the positive terminal. Their conversion into neutral lithium occurs at the positive terminal, where they are added into graphite. The movement of lithium ions converts the graphite into cobalt oxide at the negative terminal; the reaction is shown in Equation (2) *L*_*i*1-*x*_*CoO*_2_ is lithium–cobalt oxide), as follows:

(2) $$Li_{1-x}CoO_{2}+xLi^{+}+xe^{-}\Leftrightarrow LiCoO_{2}$$

**Figure 6:**
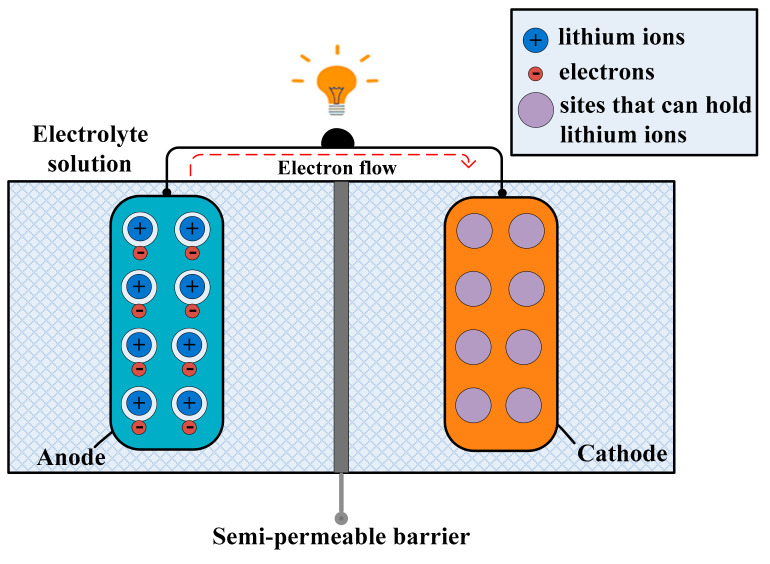
Working feature of lithium battery. Adapted from Ref. [78].

The biggest market for batteries is EV, WSNs, and low-cost microelectronic devices, and now technologies are focusing towards the main challenges of reducing the cost of batteries and increasing the range under electric power [71]. Still, LIBs are the most attractive and best option for commercial use. The energy density of these batteries has increased almost four times since 1991 [72]. According to [73], the biggest drawback of LIBs is the requirement of non-aqueous/organic liquid electrolyte for ionic transfer. The different issues that these batteries are facing are as follows: aging, capacity fading, and safety concerns. According to [74], the safety concern rose with the burning incidents of lithium batteries, and the flammability of the solvents. Due to safety concerns, the transportation of lithium batteries by passenger-cargo planes is banned. Several analyses were done for lithium-ion batteries [75,76]. For seminal novel battery chemistry and sustainability, the best raw materials, and their combination, is required. In the last few years, the usage of the Earth’s abundant metals as negative electrodes was the hottest issue. At present, LIBs are used in laptops, cell phones, and different portable devices. The requirement of an energy source in low-cost MEH and small batteries for low-power electric devices energy storage, made the development in LIBs so much more important [77]. The cathode, electrode, and electrolyte problems in LIBs could be solved by choice of good materials. When we connect a lithium-ion battery with a device, the positively charged ions move from the anode to cathode, then the cathode becomes positive as compared to the anode, as a result the electrons are attracted towards the cathode, as shown in Figure 6 [78]. The future lithium batteries are lithium–sulfur batteries. The charge holding capacity of lithium–sulfur batteries is more than LIBs. Lithium-ion batteries are very popular in low-cost MEH applications. In low-power applications, they are the best option, e.g., earbuds, earphones, mobiles, laptops, and microelectronic gadgets. 

### 3.4. Lead Acid Battery

**Figure 7:**
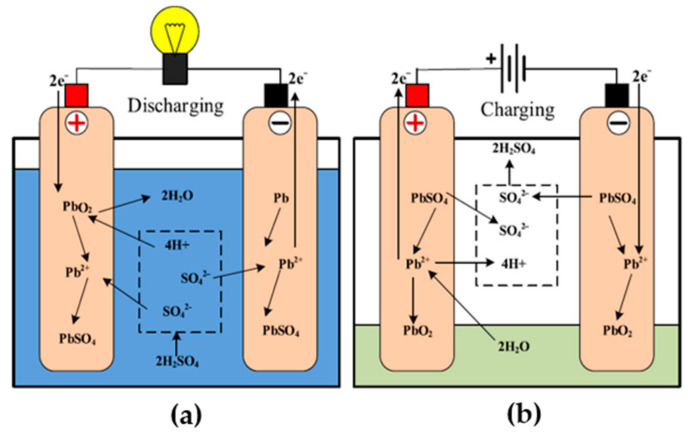
Chemistry of lead acid-based battery, (**a**) during discharging, (**b**) during charging [57].

According to [79], the application of lead-acid batteries has increased in recent years, for energy storage purposes. Figure 7 shows the structure of the lead-acid battery, consisting of staked cells dipped into a diluted solution of sulfuric acid *H*_2_*SO*_4_, a positive electrode in each cell, consisting of lead dioxide, and the negative terminal made of porous lead. At discharge, both the terminals transform to *PbSO*_4_, and return to their initial states at charging [80]. The reactions at different electrodes are explained in the equations below, during charging:

(3) $$Pb+SO_{4}^{2-}\to PbSO_{4}+2e^{-}$$

(4) $$PbO_{2}+4H^{+}+SO_{4}^{2-}+2e^{-}\to PbSO_{4}+2H_{2}O$$

(5) $$Pb+PbO_{2}+4H^{+}+2SO_{4}^{2-}\to 2PbSO_{4}+2H_{2}O$$

In these equations, *Pb* is the lead, *SO*_4_ is the sulfate, *PbO*_2_ is lead dioxide, *H*_2_*O* is the water, *e*^−^ is the electron, and *PbSO*_4_ is the lead sulfate. During charging, the lead and sulfate combine to form lead sulfate. Lead dioxide with hydrogen ions, sulfate, and electrons form lead sulfate and release water. 

**Figure 8:**
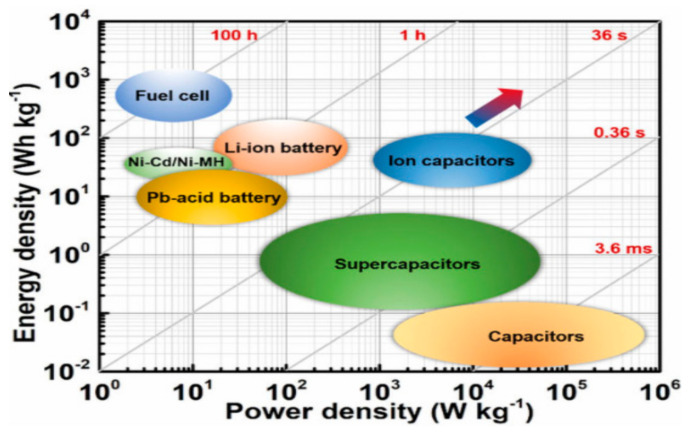
The comparison of energy density and power density for different energy storage devices. [85].

The normal voltage of lead-acid batteries is approximately 2 V/cell; for six cells, the total voltage is 12 V [81]. The battery fatherly discharges when a load is connected between its electrodes. Here, the electrodes are built of lead mixed with the alloy of 5 to 10% of antimony, which limits the positive terminal’s polarization corrosion. The positive terminal is built of a lead and lead dioxide blend. The separator is hard rubber, and the polyvinyl chloride bar avoids a short circuit, which is possible between the electrodes. The electrolyte that consists of 30 to 40% sulfuric acid, depends upon the type of lead-acid battery [82]. The main advantages of lead-acid batteries are the following: large current capacity, tolerant to abuse, tolerant to overcharging, for rechargeable cells, lower cost per unit capacity, and cheap manufacturing of the battery. The main disadvantage of lead-acid batteries is overcharging and overheating. The disadvantages include the following: the life span is typically 300 to 500 cycles, the corrosion of the electrodes can cause the burning of people and corrosion of metalwork, lead is not environmentally friendly, the acid must be treated with care, it is not suitable for fast charging, and the charging efficiency is only 70% [83,84]. The overheating and overcharging of lead-acid batteries are the main reasons for its failure. This problem can be overcome by the application of an intelligent voltage source charger. This intelligent source will first charge the battery at a constant voltage, then join later with a constant current. This source will also give protection against overcurrent, which will help to set the level of battery capacity and battery voltage. Figure 8 shows a comparison of the energy density of the batteries [85]. 

The benefit of these batteries is their low cost, high efficiency, and surge capacity [86]. The surge capacity of a device is in place to absorb the surges and reduce the steepness of the wave front. Sealed lead-acid batteries are used in toy cars, scooters, and emergency lights, etc. For WSNs, low-cost batteries are required, and replacement of the battery increases the run time of the system. Due to their low cost, lead-acid batteries are good options for WSN networks.

### 3.5. Nickel–Cadmium Battery

Ni–Cd battery cells are the mature technology of the low-cost batteries, and are famous for low-cost microelectronic devices, MEH and WSNs. The nickel–cadmium (Ni–Cd) battery contains nickel oxide, metallic cadmium as the cathode and anode, respectively, and potassium hydroxide as the electrolyte [87]. Nickel is a silver–white metal, and one of the four elements that are magnetic at room temperature. Above 355 °C it is nonmagnetic. Nickel has a crystal structure and it is hard, malleable, and ductile. Its melting point is 1453 °C, and its boiling point is 2732 °C. On the other hand, cadmium is a silver–white metal, and is very similar to zinc and mercury. Its melting point is 321 °C, and its boiling point is 766.8 °C. 

**Figure 9:**
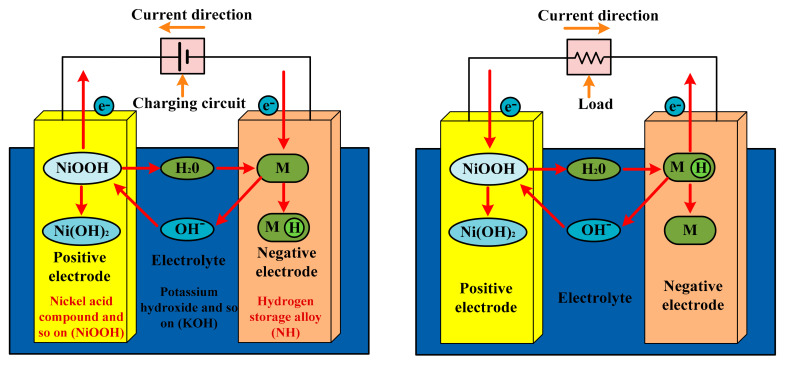
Charging and discharging of Ni–Cd battery.

The charging of Ni–Cd is explained in Figure 9, and Equations (6) and (7) [88]. Ni–Cd batteries are a developed technology, and are used in difficult environmental conditions and in places where a long service life is required [89]. These batteries have so many performance advantages. As compared to the standard alkaline batteries, these batteries have very low internal resistances. There are two important parameters of a battery that determine its performance, they are as follows: capacity and internal resistance. The operating temperature and the battery life totally depend upon the internal resistance of the battery. Due to the low internal resistance, Ni–Cd is useful where a high initial power is required for a very short duration. In piezoelectric MEH, the output power is only a few micro watts; for the storage of such a low power, a battery with low internal resistance is required, and Ni–Cd is the best available option. 

It has a good reputation for its low cost and powerfulness. On the basis of their powerfulness, these batteries can be charged at a higher rate in a short time. The main drawback of these batteries is the memory effect; they can lose capacity if they are recharged without completely discharging. In the start, Ni–Cd was preferred for medical equipment, two-way radios, video cameras, and power tools [90]. The cells with more active material achieved 60% more capacity than the standard Ni–Cd, but result in a higher internal resistance and reduced cycle count. The battery chemical equations during charging are given below:

(6) $$Ni{\left(OH\right)}_{2}+OH^{-}\to Ni{\left(OH\right)}_{3}+e^{-}$$

(7) $$Ni{\left(OH\right)}_{2}+{OH}^{-}\to NiOOH+H_{2}O+e^{-}$$

(8) $$Cd{\left(OH\right)}_{2}+{2e}^{-}\to Cd+2OH^{-}$$

In these equations, *Ni*(*OH*)_2_ is the nickel hydroxide, *OH*^−^ is the hydro-oxide, *Cd(OH)*_2_ is the cadmium hydroxide, and *e*^−^ is the electron. During charging, the nickel hydroxide and the hydroxide ion combine to form *Ni*(*OH*)_3_ and release an electron. On the other hand, cadmium hydroxide combines with electrons, and, as a result, forms cadmium and hydroxide. 

The standard Ni–Cd batteries are the most stiff and mild batteries; due to these reasons, the airline industry still uses them in microelectronic devices. However, to attain longevity, it needs care and attention. Ni–Cd and NiMH (nickel metal hydride) batteries have a memory effect that affects the capacity if the discharge is incomplete, as shown in Table 2. Actually, the battery is capable of remembering the last time energy was delivered, and once a pattern is followed, these batteries do not want to give extra energy [91,92]. Their application is in devices in which higher values of capacitance/fast discharge are important. Apart from the higher cost, the biggest drawback is the chemical components, which are poisonous. 

Ni–Cd batteries are used in portable electronic devices, toys, and garden solar lights, etc. These are very useful batteries, but they have limitations, due to memory effects, periodic maintenance, relatively high discharging as compared to other batteries, and use of toxic materials. 

### 3.6. Sodium–Sulphur Battery

**Figure 10:**
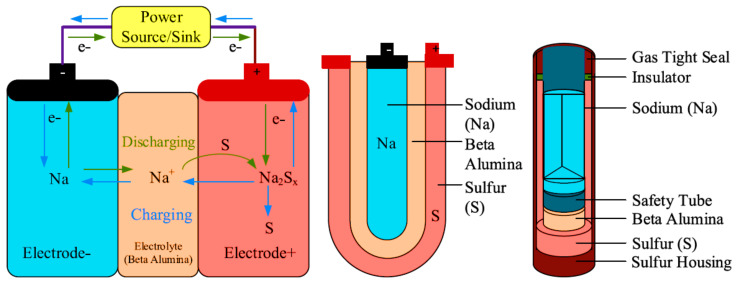
Sodium–sulfur battery [57].

The sodium–sulfur battery is a molten salt battery. Sodium is a very soft metal. It is both a mineral and electrolyte. Its melting point is 97.81 °C, and its boiling point is 882.9 °C. On the other hand, sulfur is a non-metallic chemical element, with a yellow color at room temperature. Its melting point is 115 °C, and its boiling point is 444.6 °C. The sodium–sulfur battery is shown in Figure 10. NaS consists of liquid sodium (*Na*) and sulfur (*S*). The energy density, efficiency, charge/discharge, and cycle life are high, and the cost is low, as it can be produced from cheap materials [93]. These are suitable for stationary storage applications. The temperature and highly corrosive nature of the sodium polysulfide, and the increasing size of the cells, make it more uneconomical. The working operation of the NaS battery is explained in equations, from Equation (9) to Equation (11) [94], as follows:

(9) $$2Na=2Na^{+}+2e^{-}$$

(10) $$S_{x}+2e^{-}=S_{x}^{2-}$$

(11) $$2Na+S_{x}=Na_{2}S_{x}$$

In these equations, *Na* is the sodium, *S_x_* is the sulfide, *e*^−^ is the electron, and *NaS_x_* is the sodium sulfide. The sodium with the release of electrons is converted into a sodium ion, and the same case happens with sulfite. These two combine to form sodium sulfate. 

The reverse condition happens while it is charged; the sodium polysulfide decomposes into sodium and sulfur, which return to the electrodes. The operating voltage of the cell is 2.075 V at 350 °C [95]. The operating temperature range is 300 °C to 350 °C; at this temperature, the sodium, sulfur, and polysulfide exist in liquid form, and the electrodes reactivity is increased. Therefore, NaS batteries have power and energy density values that are higher, with low cost and safety [96]. Although the NaS batteries have so many advantages over lead-acid batteries, their main drawback is maintaining the temperature between 300 and 350 °C, and the requirement of a permanent heat source that keeps the temperature within the limits. Table 3 shows a comparison between NaS and lead-acid batteries. 

### 3.7. Electrical Storage System

The microelectronic industry is one of the most important technologies. Here, the electrical energy is used, transformed, and controlled. The main applications of microelectronic devices are as follows: power supply in industrial processes, drive technology, and lighting equipment [97]. The application of the wireless systems is so much more popular these days. These systems are either powered by batteries or self-sustained, as they generate their own power by the MEH system from the ambient resources. We are living in a world of intelligent sensing. Here, countless wireless sensing devices are working every second, to provide information about weather, cropping, etc. As a result of these WSNs, the trend of this intelligent world is increasing day by day. These trillions of wireless sensors are powered by batteries that need charging after some specific time. If in the future everyone has hundreds of wireless sensors, then it would be impossible to provide the power. Researchers are not only focusing on better designs of batteries, for these sensors and microelectronic devices, but also on the designs of low-cost and highly efficient batteries for these devices. EES system technologies have the following three main functions: management, gap reduction, improvement, and reliability in electronic devices. Commercially, various EES systems are available, but they are not capable of fulfilling the requirements of an ideal EES system (i.e., mature, long life, low cost, and environmentally friendly). Supercapacitors, cells, and batteries are suitable for energy management applications. The examples of advanced batteries are micro lithium-ion and lead-acid batteries. Whereas, nickel–cadmium and supercapacitors are developed, and fuel and solar, etc., are under developed [98]. The cycle efficiency of batteries is good, whereas the supercapacitor has a cyclic efficiency of 60%. Due to the larger losses, the thermal, chemical and solar cells are facing low efficiency. The electrical system’s cyclic life hinges on the supercapacitor [45].

Thermal and mechanical EES systems that have automatic load transfer switches, and advanced thermal energy storage systems, are enjoying the advantage of long life cycles. The batteries and switch-mode power supplies are not environmentally friendly, because of combustion, magnetic field, and poisonous portion. Environmental conditions, such as temperature, affect the performance of the batteries and supercapacitor. At freezing point, the electrolyte in the batteries freezes, and at high temperatures it starts boiling. These ESS also affect the environment; for example, the emission of carbon dioxide, as a result of chemical reactions, is harmful for humans. Whereas, the solar fuel cells (low voltage), along with the supercapacitors, are environmentally friendly [99]. 

#### 3.7.1. Capacitor as Energy Storage Device

The conventional capacitors have the ability to stock energy in electrical charge form. They produce voltage over the plates, which makes them similar to a small re-energized battery. Different types of capacitors are used as part of resonance circuits or in power factor corrections, but all of them perform the same job of storing energy. A simple capacitor comprises two conducting plates made up of metal, electrically separated by insulating material (waxed paper, mica, ceramic, etc.). The insulation between the plates is known as a dielectric. Generally, capacitors are not preferred to store and provide a large amount of energy, as their energy density is less as compared to the batteries. However, they are very useful to fulfill short-duration power requirements, as their charging/capability is very high as compared to the batteries. The maximum operating temperature of capacitors is 105 °C, and their lifetime is as little as 1000 h to 10,000 h, or more [100]. 

**Figure 11:**
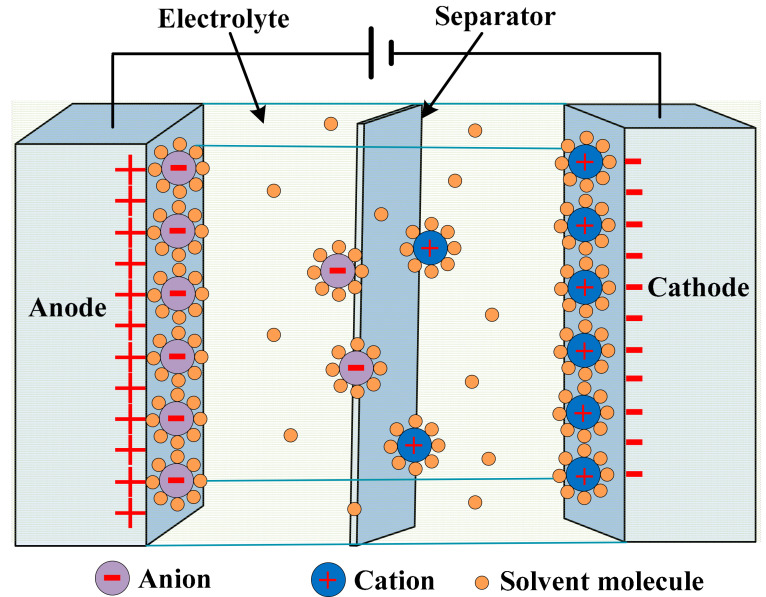
Capacitor as storage device in micro-energy harvesting.

The electrolytic capacitors have many technical problems, such as failure at high temperatures, internal power dissipation due to ripple, reverse voltage, and transient voltage. By means of static charge, the energy is stored in a capacitor, opposing electrochemical reaction. By the application of a voltage between the positive and negative plates, the capacitors are charged. Capacitors have three categories, the most basic is known as an electrostatic capacitor [101]. In this type, a dry separator is used, having very low capacitance and being capable of filtering and tuning radio frequencies. The electrostatic capacitor gives higher capacitance than electrolytic capacitors. These capacitors use a moist separator, and are utilized for filtering, signal coupling, and buffering. The third type is the supercapacitor, the best application of these capacitors is energy storing, where frequent charge/discharge is required at a high current for short durations [102,103]. Despite the continual research of electrolyte capacitors, the major disadvantages still remain, including power loss. This disadvantage in some systems can affect the performance of the entire system. In Figure 11, the energy that is generated by the piezoelectric energy harvester is stored in a capacitor [104]. The electrical energy that is generated by the piezoelectric transducers is very low. It is rectified, increases in voltage by a boost converter, and is finally stored in a capacitor. It is clear from Table 4 that the capacitor and batteries have different advantages, and if the battery has the advantage of low cost and a stable voltage, then the capacitor has the advantage of fast charging/discharging ability. 

#### 3.7.2. Ultra-Capacitors

The ultra-supercapacitor is a double-layer capacitor with high capacitance. Supercapacitors have two key storage theories, as follows: 

Double-layer capacitance: The storage that is achieved by the forces of charges over the Helmholtz double layer between the electrolyte and exterior of the electrode. The charge gap is 0.3–0.8 nm, which is much smaller than conventional capacitors;

Pseudo capacitance: The energy that is stored by the Faradic process. The charge transfer starts with a redox reaction, which is reversible electro-absorption [105]. Electrostatic double-layer supercapacitors (EDLCs) do not have a conventional dielectric. Instead of an intervening insulator between two plates, these capacitors have virtual plates that have two similar films. The electrochemical properties of the electrical double layers results in the active separation of charges, instead of vanishingly thin physical separation of the layers [106]. The smaller capacitance is due to the lack of need for a bulky layer of dielectric and porosity of the materials that are used to permit the packaging of plates in a much larger area. The electrical double layers are conductive, but no current flows between them [107]. 

Each EDLC consists of the following two electrodes: separator and electrolyte. A metallic foil collector connects the two electrodes to the terminals. Generally, electrodes are made up of activated carbon, as this material is capable of electrical conductivity and high capacitance, due to its larger surface area. The electrodes are separated by a medium (ion-permeable membrane) to prevent the short circuit between them [108]. The composite can be folded in rectangular/cylindrical shape or can be stacked into a rectangular housing or in aluminum. The cell is suffused with viscous or liquid electrolyte (organic/aqueous). This electrolyte depends upon the application, peak current demand or power requirement, allowable temperature range, and the operating voltage. It is sealed from the outside. Mostly EDLCs are constructed with two electrodes and a separator [109]. 

**Figure 12:**
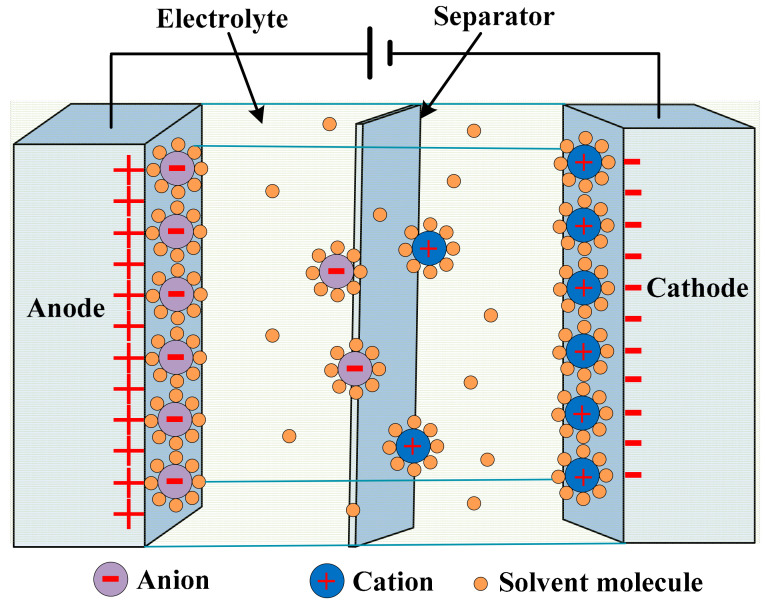
Structure of electrostatic EDLCs.

By the application of voltage, the ions in the electrolyte disperse over. The dispersion is in the holes of the opposite electrodes. The charged layers formed due to the disassociation of hundreds of angstroms between the electrodes and electrolyte, as shown in Figure 12 [110,111]. The charge separation is caused by the double-layer capacitance [112]. High capacitance values are achieved, as it increases with increases in the surface area, while it decreases with increases in the distance between the layers. 

Supercapacitors gained so much more attention than batteries, as they have a fast storage capacity (shorter discharge time supercapacitor: 1–10 s vs. lithium-ion battery: 10–60 min) and increased cyclic stability (supercapacitor > 30,000 h vs. battery > 500 h) [113]. A supercapacitor is a burst current system. Its specific power is high (10,000 Wkg^−1^) and current for a short duration, i.e., less than 1 min. As a result, supercapacitors can be used alone or in fusion with batteries, which improves the efficiency and increases the cyclic life for operating MEH, WSNs, and low-cost microelectronic devices [114]. Now, the substantial development in supercapacitor technology is to increase the electrical importance of the electrode material. Different types of supercapacitors are used in drive systems, for starting and recovering braking energy [115]. Consequently, supercapacitors are the best options for energy storage applications, e.g., backup power supply system against power disruptions [116]. The efficiency of supercapacitors is affected by many factors, such as the electrolyte, electrode material, and potential window of electrodes [117]. 

**Figure 13:**
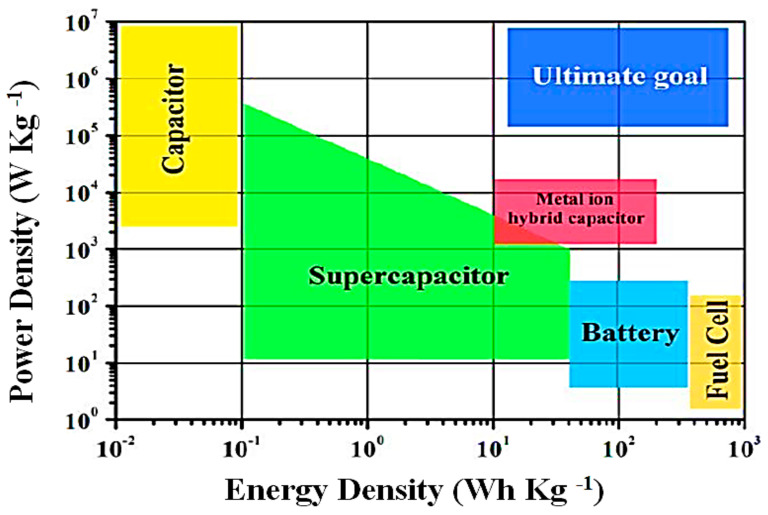
Ragone plot for different storage devices. [121].

Now, supercapacitors have gained popularity in different energy fields, where constant high energy (toy cars, toy drones) and constant stable energy (computer chips, portable electronic devices and WSNs) are required [118]. The use of supercapacitors is only restricted in backup devices because they cannot store the maximum energy. Supercapacitors form a bridge between batteries and vintage capacitors by overcoming the problems of cell voltage, specific power, and operating cost. The traditional capacitors are preferred, where storage and quick power release is required, and power delivery and liftoff of 196 k Wkg^−1^ occurs in just a few seconds [119]. On the other hand, supercapacitors have higher specific energy (10.1 Wh/kg) as compared to electrostatic capacitors (6 Wh/kg) [120]. The supercapacitor has alluring features, such as higher values of movement of energy per unit area and a brisk charge/discharge procedure; this is also a reliable constant source of power. In Figure 13, the exchange of power density and energy is described graphically [121].

In this figure, the power density of electrostatic capacitors is high with a low energy density, fuel cells have the highest energy density with a low power density, and the best option is electrochemical capacitors with better values of power and energy densities. The Ragone plot is very useful for estimating energy storage performance, but ignores the critical features, such as cycle life, cost estimation, and safety for comparison between supercapacitors and batteries. For a better comparison and understanding of energy storage technology, it is important to consider safety, cost estimation, and cycle life. In supercapacitors, carbon nano-tubes, carbon nanofibers, and graphene are used. The main disadvantage of carbon nanotubes is the lack of solubility in aqueous media. The carbon nanofibers are toxic and graphene is bad for humans and the environment. Better electrode materials are required for the improvement of supercapacitors. The main difference in batteries and supercapacitors is the storage formula, energy storage amount, power restrictions, and life [122]. 

The cyclic life of a supercapacitor is 1 million to 30,000 h, i.e., extraordinarily higher than the batteries, which are 500 h, and the recharging time for the supercapacitor is remarkably low, i.e., 1–10 s vs. battery 10–60 min [123]. Some advantages and disadvantages associated with supercapacitors are mentioned in Table 5, with some applications. The disadvantages must be overcome to make them ideal for use in the energy storage industry. This comparison explains that the storage mechanism in supercapacitors is not a reversible chemical reaction, and it can withstand half a million cycles. Among the different characteristics that are important to explain the performance of energy deposit devices, an important one is temperature range. One of the most attractive features of a supercapacitor is thermal stability, which is tuned by a wise choice of materials, including electrolytes. The operating temperature of the battery is between −20 and 60 °C, and that of the supercapacitor is between −40 and 100 °C. According to [124,125], several efforts have increased the device capacitance, such as (a) new materials having a large surface, (b) upgrading splash electrode facet, (c) adjusting the mass transport mechanism, and (d) inventive/active materials that allow high mass transference. 

### 3.8. Chemical Energy Storage Systems

The chemical ESS is an important energy storage system, in terms of its long-term storage in the form of chemical bonds of molecular compounds. Power is produced as a result of chemical reaction, due to the rearrangement of the molecules through electron transfer. The primary substance is converted into a new energy form. Chemical ESS is an environmentally friendly system, with zero-emission mechanics, having a storage capacity from low- to high- voltage levels [126]. In the chemical ESS, the chemical energy fuel cell (FC) provides continuous electrical energy generation (0.5 to 1 V). As compared to battery systems, the FC will supply the electric power continuously until external oxidant and fuel are available; harmful gas emissions are reduced by decreasing the dependency on fossil fuels [127]. 

**Figure 14:**
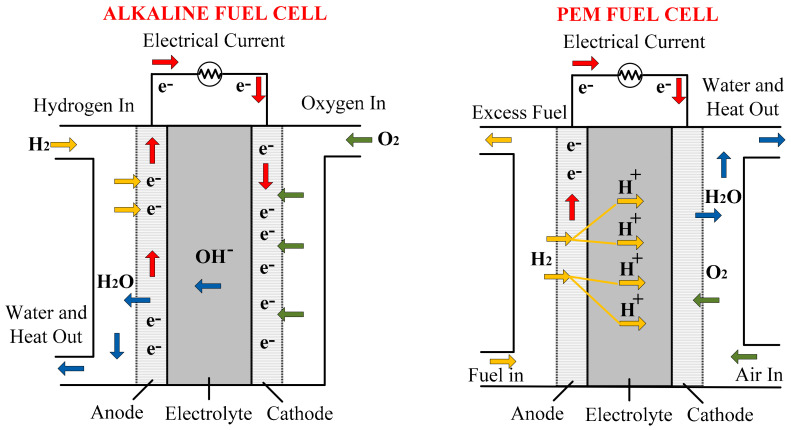
Alkaline fuel cell internal structure.

The chemical energy storage system consists of batteries and fuel cells (using fuels from renewable resources). From mega- to micro-level applications, batteries are very popular for electrochemical energy storage. Chemical batteries are a promising source of power in microelectronic devices, portable electronic devices, such as cell phones, laptops, toys, etc. For portable electronic devices, which need a low energy density, the lithium-ion batteries have a greater energy density and discharging time than other batteries. This is the main reason for using different chemicals in these batteries, to increase their efficiency and charging/discharging time. In Figure 14, the construction of an alkaline battery cell is explained [128]. The construction is very simple, containing hollow nickel-plated steel canes filled with manganese dioxide, graphene, and potassium. The combination is the anode and the steel acts as the cathode, and a fiber or a paper is dipped in the electrolyte and placed inside the cane. A gel containing zinc powder and potassium hydroxide is placed between the separators, in order to make space for chemical reaction in the cell after use. A current collector (brass pin) is inserted into the center of the cell. There is a plastic seal with a metal end-cap, and the current collator is welded with this cap. A steel plate is used to seal the other end of the cell.

### 3.9. Thermal Storage Systems

Thermal energy storage (TES) is an energy storage technology that absorbs the thermal energy by heating or cooling a storage medium, and this stored energy can be used later to produce a power source, or for heating or cooling in some applications [129,130]. TES are widely used in buildings and industrial processes. TES is mostly used to capture the heat energy of the sun and reduce the energy requirements of the buildings. The recent projections are predicting that, until 2040, the primary energy demand will be increased by 48%. On the other hand, due to the excessive use of fossil fuels and their unfriendly environmental impact, the world is turning towards renewable energy resources. Nowadays, renewable energy resources, such as vibrational energy, solar, wind, ocean waves, and bio fuels, are satisfying the global power demands, while maintaining the natural balance. However, due to the expected increase in power energy demands and the unexpected climate changes globally, the most urgent issue to be addressed is to store the energy of renewable energy resources with a better storage technology, which should be efficient and use a sustainable method. In the MEH system and WSNs, the energy storage has become an integral part of the system. 

The thermal energy of the human body depends upon the activities of the human body and the environmental conditions. The human body’s thermal energy ranges from 290 to 3800 kilojoules per hour, which is equivalent to 80 to 1050 W. In the recent decades, the idea of converting this unusable energy into usable energy has gained so much attention. Generally, a human male generates 100 to 120 W. A small amount of this unusable energy can be utilized by the thermoelectric device to operate electronic devices. 

Thermal energy storage technology is widely used around the world; in 2017, the world’s thermal ESS capacity was 3.3 GW, which was 1.9% of the world’s energy deposit [131]. Thermal ESS systems can provide load shifting along with heating/cooling in domestic/industrial sectors, hence they play an important role in the demand management of micro grids [132]. The thermal ESS is incorporated by the following three main parts: thermal storage container, heat transfer mechanics, and control system. First, attainable heat is stored in a storage reservoir, by the application of different technologies. The heat transfer method is used directly/indirectly to extract the stored heat energy with the help of a combustion engine. The containment control system is used to monitor the insulation, heat transfer medium and operation of the storage reservoir [133]. The main disadvantage of thermal ESS is the low cycle efficiency, at 30–50%, which is overcome by advantages such as energy density and self-discharging 1% per day, low capital cost, together with environment friendliness. This makes thermal ESS the best choice for different applications. 

There are two types of thermal ESS, depending on the temperature and energy storage materials, i.e., thermal ESS with small and large values of temperature [134]. Thermal ESS with smaller values of temperature contain cryogenic energy storage and auriferous low-temperature storage systems. In auriferous low temperatures, thermal ESS water bathing as well as reheating is used, while in cryogenic energy storage thermal ESS, liquid air/nitrogen is used.

For high power density applications, the thermal ESS with low temperatures is suitable, such as industrial cooling, load shaving, and power management in the future micro grids. In high-temperature thermal ESS, latent heat and sensible heat systems are important. In sensible heat ESS, thermal energy deposits with temperature alterations in the depositing material, provided there is no change in the material. The capacity of a heat storage system depends on the mass, along with the specific heat. Storage systems have different masses in different states, for example liquid (water), solid (concrete), etc. [135]. A major problem with a sensible heat deposit system is the requirement of a large amount of space. 

In latent heat deposit, energy is reciprocated when the material changes through metal matrix structures or metallic filters [136]. Phase change materials (PCMs) have three types, based on different combinations of materials, as follows: solid–solid, solid–liquid and liquid–gas. For thermal ESS, the most suitable PCMs are solid–liquid. Solid–liquid PCMs avail, organic, inorganic and mixture of materials. 

Equation (12) presents the deposit of energy in a mass.

(12) $$Q=m\left[C_{PS}(T_{m}-T_{S})+h+C_{PL}(T_{l}-T_{m})\right]$$

Here, *T_m_* is the melting temperature, *T_S_* is the solid temperature, *T_l_* is the liquid temperature, *m* is the mass, *C_PS_* is the convective heat transfer coefficient, *h* is the solubility coefficient, and *C_PL_* is the capillary pump loop. Equation (12) shows that the energy deposit in a mass depends upon the temperature of the mass in different states, solubility coefficient, convective heat transfer, and capillary coefficient of the pump loop. 

In a system, the storage capacity is always decided by the PCMs melting temperature and phase change. They are also known as “invisible heat deposit systems”, because the rate of variation is zero during the energy trade [137]. The old version of heat systems possesses a high energy density, which is economical and more efficient when compared with sensible heat systems. Consequently, they are easily adoptable in dispense energy resources when used in buildings. The power plants, which use solar energy, use two deposits. Each container deposits the thermal ESS of the sun, and the temperatures of two containers are 500 °C and 285 °C [138]. In the future, smart grids will shift towards the thermal ESS methods, where the cost is low and the temperature is high. 

### 3.10. Hybrid Storage Systems

Hybrid ESS is the effort to create a device that has the qualities of different energy storing devices. Individual ESS cannot have all the desired technicalities, such as power rating, energy density, operating temperature, life cycle and discharge rate, bearing the difficult working environment in real-time applications [139]. In hybrid ESS, the improved cycle efficiency and optimal performance is achieved by coordinating the qualities of the available ESS [139]. The purpose of all the hybrid ESS is to merge high energy density with high power densities in devices, to achieve power and energy rating characteristics [140]. 

The possible combinations and size of the hybrid ESS depends upon the factors of economy, environment, and application [141]. For controlling the problems of low efficiency and increased cost of DC converters, a capacitor has been proposed, which is capable of the qualities of more than two devices. As compared to the old one, hybrid capacitors have unique capacitance [142]. This is the blend of batteries and capacitors features, with overall increased potential, power densities, and energy densities. The hybrid capacitor shows a specified energy of 35 Wh/kg. Whereas, it shows a power density of 1000 W/kg [143,144]. The operation temperature in hybrid capacitors ranges from −50 °C to +200 °C at 5 KHz frequency. The supercapacitors are used in WSNs, MEH, power stabilization, regenerative braking, catalyst preheating, and low-cost microelectronic devices [145]. The li-ion blend capacitors look more attractive among different material combinations. They are the best option in overcoming the differences in lithium batteries and high power density devices [146]. They are the blend of lithium in ionic form, with carbon used as the cathode and penetrable triggered carbon materials used as the anode [147,148]. In an aluminum plastic bag, a stack is formed, in which a capacitor with a lithium sheet is slotted.

**Figure 15:**
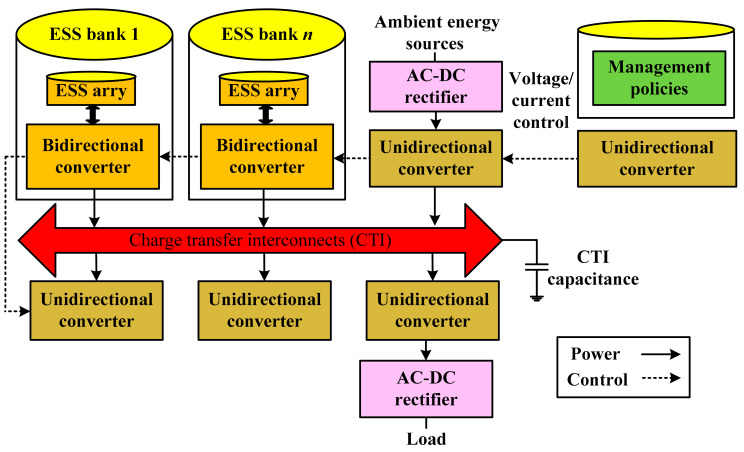
Generalized architecture of hybrid energy storage system.

In electrochemical discharge, the lithium metal foil is introduced as an alternate electrode to attain carbon anode pre-doping [149]. The reaction rate of the hybrid capacitor is 98%, with stable efficiency [150]. The difficulties of pre-doping are the changing voltage, establishing the interphase layer, and the high rate metal plating [151]. Na-ion hybrid capacitors are an alternative economic, and easily available, model [152]. Hybrid ESS are shown in Figure 15. 

This architecture ensures the stability, modularity, flexibility, and scalability of hybrid systems [153]. Here, a medium is used for charge transfer, especially for the interconnection between the different hybrid ESS, known as charge transfer interconnection (CTI). All the hybrid ESS banks contain many comparable ESS elements. A bidirectional converter is present to help these elements match the voltage in CTI during charging/discharging. Different load devices and power sources are connected to hybrid ESS through CTI and a unidirectional power converter. Depending upon CTI topologies, hybrid ESS can be categorized as active topology (decoupled connections hybrid ESS banks according to their characteristics), passive topology (the hybrid ESS–bank connections are parallel), and discrete topology (reconfigurable adaptive switching topology) [154,155]. 

The charge management policies and system controller are tools to control and optimize the operation mode, connections, current, and potential during charge and discharge, through current replacement of CTI [156]. The best performance of hybrid ESS at each instant is achieved by controlling and monitoring the status of each ESS bank. As is clear from the figure, it consists of a micro harvested energy storage system. After the completion of the load requirements, extra energy is stored in the form of kinetic-energy, electrical energy (energy dispatch system), thermal energy, which is in the oil tanks, and mechanical energy. The stored energy will overcome the electricity gap when abandoned energy is not available, e.g., unstable vibrations in piezo micro-energy harvesting, wind deficit times or peak loads; this is achieved by a control system. Therefore, the use of versatile hybrid ESS is the optimum option in the future, to solve the problems that are related to individual storage systems; it requires research development and interdisciplinary collaboration for future developments, to attain progress in this field [157]. 

## 4. Comparisons of Energy Storage Technology

ESS is the important component to solve the problems of storage in low-cost microelectronic devices, WSNs, and MEH. For the efficient design and control of ESS, the comprehensive investigation and analysis of ESS versatility, in terms of physical constraints, technical specifications and their effect on nature, must be known [158]. The system design and preferences are measured in terms of storage duration, standby time, ESS maturity, response time, life cycles, storage losses, economy of storage, conversion efficiency, thermal grading, safety concerns, compatibility to automation, and usage purpose, along with environmental impact [159,160]. In a single ESS, there is a rare possibility of fulfillment of all these characteristics simultaneously; therefore, based upon the maximum storage discharge time and capacity requirements, we can exceed the optimum suitable ESS technology. 

ESS adaptation capital cost is affected by the mass production and technical maturity as Na–S, Li-ion, and Ni–Cd, along with TES developed, commercially. The TES system is low in efficiency, due to its high conversion losses [161]. The application of ESS is classified on the basis of charge/discharge time, power grading, and electrical system to the consumer level. In Table 6, different the battery features that are required for different electronic devices are listed. 

### Supercapacitor vs. Battery

Nowadays, the most promising energy storage device in MEH is the supercapacitor. The long-term reliability, high power density, and high energy features, makes the supercapacitor applicable for the auxiliary power unit, backup power unit, along with power compensation [162]. Batteries have 500–1000 charging/discharging cycles, while supercapacitors can reach up to one million cycles. In WSNs and MEH, the batteries have a two-year life, while supercapacitors could last for 10–15 years. The charging time of a supercapacitor is 1–10 s. The charge characteristics of a supercapacitor are similar, but the charge–current is limited by the charger’s current holding capacity [163]. In MEH systems, e.g., vibrational energy harvesting and powering wireless sensors in IoT at remote places, supercapacitors are the best option. Table 7 shows the comparison between batteries and supercapacitors, and it is clear that the supercapacitors are better than the batteries in many ways. 

In supercapacitors, the potential energy deposits in the electric field, whereas batteries store potential energy in the form of chemical energy. The batteries provide a higher energy density for the storage of power, while the supercapacitors have a faster charging and discharging capability, which makes them more attractive than the batteries. 

Supercapacitors are designed to provide (a) high power, (b) long stability, and (c) efficient storage, because the electrodes are highly absorbable materials. However, their disadvantages, such as low energy density as well as specific capacitance, makes them unfit for applications where energy is required long term [164]. The use of a scalable nano-porous graphene synthesis mechanism involves heating hydrogen; the graphene supercapacitors have a quality of energy density, better than the lithium batteries [165]. The supercapacitor is made up of spongy and porous material, with an extraordinarily high specific area. The smaller pores of the plates increase the ESR, which finally decreases the specific power. 

**Table 7:** Batteries and supercapacitors comparison on the basis of favorable and unfavorable conditions.

Battery Unfavaourable Condition	Supercapacitor Unfavaourable Condition	Improvement	Analysis	Reference
Battery is stressed during peak demand conditions in electronic equipments.	Not stressed during peak demand conditions.	Supercapacitor should be connected in parallel with battery in microelectronic devices, WSNs to enhance the battery life.	The hybrid combination of supercapacitor, battery and inverter has improved efficiency and performance of the energy storage unit.	[116]
Battery has a finite lifespan; replacement on discharging in hilly areas is difficult.	Supercapacitor is a good option.	Hybrid combination increases the life of ESS.	Piezoelectric energy harvester is a good replacement for battery in remote locations.	[166]
i. Lead acid batteries are capable of long cycles and cylic life. ii.Li-ion batteries have advantages of energy density and specific energy, less important for static installations.	Supercapacitor in hybrid construction improves shallow cycle performance.	Some types of lead-acid batteries have hybrid construction with a supercapacitor element with conventional negative plate.	Only lead-acid batteries are good storage options among batteries and capable of complete recycling.	[167]
Conventional lead-acid batteries are the weakest link in photovoltaic installations.	Supercapacitors are a good option.	Hybrid valve regulated lead-acid batteries have (i) Lower internal-resistance (ii) High thermal stability.	Hybrid valve-regulated lead-acid battery performance is better.	[168]
In lead-acid batteriesmechanical stress on the charging/discharging cycle weakens the active material and causes softened corrosion and cracking of grids. The irreversible sulfation of the plates and internal short circuit damages the battery.	Important for micro electronic devices. Supercapacitors have high energy density, quick charge/discharge process.	The design can be modified by including glass fiber mats around the positive plates and using thicker positive grids. Flexible solid state supercapacitors have high power density.	Deep cycling ability is achieved.	[169]
			The fabrication of capacitors with Na^+^. Advantages of energy-density along with stability.	[169]
Replacement of batteries is problematic.	Deliver high-power (10 k Wkg^−1^) release in a very short time.	Nano engineering improves the capacitance of the supercapacitors and attains high energy density.	Improve the energy density by increasing the working voltage window by using a stable electronic electrolyte.	[102]
Lithium ion batteries cannot discharge at large currents. lithium–sulfur batteries have low stability. Sodium ion batteries have worse electrochemical performance.	(i) Most efficient storage device (ii) High power density (iii) High energy density (iii) Long cycle life (iv) Fast charging discharging (v) Instant high current discharge (vi) Low cost (vii) Easy maintenance and (viii) No pollution.	Wise choice of electrode materials can improve the performance and reduce the cost.	Supercapacitors are green-devices. They are efficient.	[170]
Volume and long charging time is problematic. Advantages still lack.	In piezo energy harvester (PEH) if a regulator is used in a charging capacitor, the charging time is increased.	If in PEH a voltage regulator used for constant voltage to charge a supercapacitor, must be avoided as it increase charging time.	The main disadvantage of supercapacitors is low energy-density.	[171]
Reduced life.	Small voltage requirement for capacitor. Fast charge/discharge.	If the energy density of supercapacitors is improved they are ideal as energy storage devices.	Capacitors ideal for storage of energy for short duration.	[172]
Battery technology has several disadvantages (i) weight, (ii) volume, (iii) large internal resistance, (iv) poor power-density and (iv) transient response.	Supercapacitor most-reliable	With hybrid models better results can be achieved.	Batteries facing the limitation, reduce the power levels. However, supercapacitor deals with different power-levels.	[173]
Battery life is small.	Supercapacitors had resolved the limitations of lead-acid batteries and proved excellent power performance.	The energy density and voltage of the supercapacitor must be increased.	Rapid change in the power such as acceleration, regenerative braking and efficiency at low temperature all these problems can be solved by supercapacitors.	[174]

The reliability of a supercapacitor is performing its function under given operating conditions and environments for a specific length of time. The reliability of supercapacitors is higher than batteries; batteries have 500–1000 charge/discharge cycles, while the supercapacitor can last for 5 to 10 years [175]. 

The supercapacitor fully charges within 1–10 s. The charging characteristics of a supercapacitor are similar to that of an electrochemical battery. The charging current is limited by the chargers current handling capability [176]. The initial charging is very fast, but the topping charging takes extra time. In constant current charging, a supercapacitor holds a voltage that rises linearly with time. During this constant current charging phase, the chargers typically monitor the output current, influencing the voltage across an external sensor (resistor) [118].

The life time of supercapacitors is more than batteries. Even though, the initial cost of the supercapacitors is very high, almost $2400–$6000 per kilowatt-hour for energy storage, and the lithium-ion batteries are used for electric vehicles, with an initial cost $500 to $1000 per kWh; although the initial cost of supercapacitors high, in long term the supercapacitors are cheaper/comparable. 

## 5. Challenges and Issues of Supercapacitors and Batteries

Supercapacitors are used in applications requiring fast charging/discharging cycles, automobiles, trains, and cranes. Here, they perform regenerative braking, burst-mode, or short-term energy storage. On the other hand, batteries can store voltages a hundred times more than capacitors. These storing devices are facing some challenges, which are given below:

### 5.1. Technical Problems

The energy density of supercapacitors is not high, and forms a gap in terms of the energy densities between supercapacitors and batteries. The low energy density can be increased by enhancing the electrode’s surface area of EDLCs. The charging and discharging cycles of supercapacitors are more than batteries. The reason for the low power density of batteries is because the chemical reaction takes time to release electrons. 

### 5.2. Establishment of Electrical Parameters Model

Generally, in many cases, the ideal capacitor model can be applied for a designing purpose in low-cost microelectronic devices, WSNs, and MEH systems, but electronic military applications, and especially satellite power supply applications and spacecraft, include some realistic parameters, which involve a potential risk, which cannot be neglected. Supercapacitors are capable of instant throughput, as a result of their high energy ability. So, in designing, the associated effects of (i) load nature, (ii) load fluctuation, (iii) external environment, and (iv) accidental impact on system stability cannot be ignored [177]. There are challenges in the design of the power density, energy density, cost, and life of batteries. For batteries, the best model is that which meets these challenges. 

### 5.3. Consistency Detection

The supercapacitor has a very low rated voltage (2.7 V), for high voltages requiring series connections to be implemented physically. Application in high current charging/discharging and overcharging can reduce the life of supercapacitors. The consistency of batteries depends upon the following two factors: SOC (initial state of circuit) and temperature. The performance of lithium-ion batteries depends upon the consistency of the battery. The performance of lithium batteries can be improved by partial discharge cycles, by limiting the temperature of the battery, and avoiding deep discharging below 2 V. 

### 5.4. Industrial Standards

Enterprises engaged supercapacitors due to their short development time, along with their fast speed. The healthy development of supercapacitors as storage devices is not possible without the supervision of the industry, with the aim of practical standards. Batteries are the integral part of so many industries, and their performance, strength and efficiency is set according to industrial standards. So, it is important to set standards for (i) model naming method, (ii) terms, (iii) electrical performance methods, (iv) general specifications, (v) safety technical requirements, (vi) electrode material specifications, (vii) electrolyte specification, (viii) charger specification series, and (ix) production technical requirements, along with (x) transportation [178]. 

## 6. Conclusions and Recommendations

This review delivers a broad analysis and information about ESS for low-cost microelectronic devices, WSNs, and MEH systems. As the first contribution, the review explains the chargeable batteries, and gives a detailed comparison among batteries and supercapacitors, based on power densities, energy densities, operating temperatures, voltages and their efficiencies. The second contribution is, it explores different energy storage technologies for ESS. The chemical energy storage and thermal energy storage systems (used in batteries) are discussed, each energy storage technology has its own advantages and pros associated with it. 

The ESS is affected by the power demand, but other vital problems, such as sources, cost, maintenance, and climate change, also play an important role. To solve these problems in power grids and low-cost microelectronic devices, new and sustainable solutions, such as renewable energy resources, have started to be implemented. The random behavior of renewable energy resources requires additional systems for such resources. The future of low-cost ESS depends upon abundant energy resources. The piezoelectric energy harvesting system (low-cost MEH system) is analyzed for powering WSNs sensors. The third contribution is the analysis of low-cost battery-based ESS. This review paper focuses on battery energy storage systems that have many problems, such as cost, replacement in the case of charging/discharging, volume, size, risk of explosion, and toxic and acidic materials, such as electrolyte, etc. These problems make them a non-reliable energy storage option. The fourth contribution is the detailed discussion on the technical issues and remedies for supercapacitors and batteries. The two main challenges of the supercapacitor are energy density along with cost, which must be overcome without losing the exponential rate performance and high cycle life. On the other hand, the batteries have the challenge of cyclic life, and fast charging and discharging. A hybrid combination of the supercapacitor and battery is considered a better option for electrical energy storage. However, in a hybrid system, energy management is a problem. This problem can be overcome by an energy management system that increases the battery life, system efficiency, and exploits the supercapacitor characteristics. A perfect topology is also required, to manage the power flow between the supercapacitor and battery. There are a few suggestions for the improvement, as follows:

- The supercapacitors store a small energy of power. To overcome this problem, the hybrid combination of supercapacitors with the lithium-ion battery is ideal, which not only improves the power capacity, but also provides a high power density, energy density, and efficiency of the ESS;
- The ESS performance is also affected by substandard terminals and electrolytes. The use of new materials in the manufacturing of electrodes and electrolytes shows better results in batteries and supercapacitors. For the construction of new electrodes, the combination of different materials, such as polymers, metal oxides, and carbon materials, is suggested. For supercapacitor electrolytes, new electrolytes are introduced, which are a combination of aqueous and non-aqueous electrolytes. There is also an active way to increase the voltage level in aqueous electrolytes, with the design of an asymmetric supercapacitor. The voltage level will increase above the thermodynamic limit of 1.2 V, and, as a result, the energy density will also increase;
- The batteries and supercapacitors are affected by environmental conditions, such as temperature and humidity. Corrosion is a big problem for the terminals. The emission of carbon dioxide affects the environment;
- For the low-cost microelectronic devices, the size of ESS is an important feature. In these systems, nano-electrodes are suggested. These electrodes have the additional advantages of a faster transient response, increased mass transport, and reduced destructive probes. In supercapacitors, carbon nano-materials and silicon nano-wires are used as electrodes, but PANI (polyaniline) is the best electrode material. The combination of PANI with graphene oxide is best for future applications;
- Electrochemical behavior and flexibility are the main problems in supercapacitors. The carbon electrodes 3D and 4D supercapacitor structures are suggested for portable and wearable electronic devices;
- The future of supercapacitor electrodes is the fabrication with the waste materials, but a lot of research is required to gain the best results;
- The batteries face stress during peak demand conditions in electronic equipment. The best solution to this is the hybrid ESS, containing supercapacitors and batteries.

## Figures and Tables

**Table 1:** Battery deterioration and failure processes.

Battery Deterioration and Failure Processes	Battery Features
Reduction in the availability of electrochemical reaction due to densification of active materials with loss of porosity.	Over a good depth discharge stable voltage.
Shedding and expansion of active material from the electrode materials.	High energy density and high specific density, made of easily available and inexpensive material.
At the negative electrode, the growth of metallic needles causes an internal short circuit.	High energy efficiency, maximum recycling efficiency.
Overcharging causes gassing of electrodes, affects battery performance.	Wide operating temperature, ability to work properly under overcharge/discharge.
Battery performance is affected by parasitic reactions.	On open circuit, maintain the charge, rugged, abuse-resistant, maintenance-free, safe in normal and abnormal conditions.

**Table 2:** Advantages and limitations of Ni–Cd batteries.

Advantages	Durable and has a large life cycles if properly maintained
	Only battery with ultra-fast charging with little stress
	Good load performance; can bear rough handling
	Long shelf life
	Simple storage
	Low temperature does not affect the performance
	Low cost
	Availability in different sizes and performance options
Disadvantages	The specific energy is low as compared to hybrid systems
	Memory effect must be fully discharged before recharged
	Due to the toxic nature of cadmium metal cannot be disposed of in dumping grounds
	High self-discharging
	The cell voltage is very low, only 1.2 V, to achieve high voltages a large number of cells are required

**Table 3:** Comparison between NaS and Li-acid batteries.

Sodium Sulphur Battery	Lead-Acid Battery
Specific energy density 760 Wh/kg at 350 °C, three times greater than lead-acid battery.	Energy density is three times less than sodium sulfur battery.
Less than half the space required as compared to lead-acid batteries in commercial applications.	More space required in commercial applications.
No self-discharge.	The self-discharge rate is approximately 4% per week.
100% coulombic efficiency (also called current efficiency, by which charges are transferred in the battery).	90% coulombic efficiency.
No intermediate reaction, 85% average DC conversion efficiency.	80% DC conversion efficiency.
No need for pumps or valves.	Can use a valve for gas blow off.
Maintenance is required after periodic inspection and cleaning.	Maintenance is required after periodic inspection and cleaning.
Environmental friendly, sealed properly, no risk of explosion during operation.	Not environmentally friendly.
99% recycled.	Each part of the old batteries is recycled.

**Table 4:** Comparison between batteries and capacitor considering temperature, charging/discharging time, and voltage.

Capacitor	Battery
Electric field for storage	The chemical reaction for storage
Submissive component	Active component
Energy-density low	Energy-density is high
Charging/discharging fast	Charging/discharging slow
Provide unstable voltage	Provide constant voltage
Operating temperature range is −3 °C to +125 °C	20 °C to 30 °C during charging and 15 °C to 25 °C during discharging
Higher cost	Low cost
Contrive of metal sheets	Contrive of metals, chemicals

**Table 5:** Advantages, disadvantages and applications of super/ultra-capacitors.

Advantages	Disadvantages	Applications
Super-fast rate of charging and discharging	They store a smaller amount of energy than a battery does.	Energy harvesting
Life spans 500,000 plus charge/recharge cycles	Faster time to discharge.	Railways
Capacitance greater than 1000 F at 1.2 V	Highly trained persons required to operate.	Charging laptops
A good option for WSN’s sensors that need peak currents.	Cost is relatively higher than batteries.	WSN’s sensors
High energy density, specific energy and cyclic life.	Cost is relatively higher than batteries.	Micro-energy harvesting

**Table 6:** Specific application and battery features.

Application	Required Features	Energy Storage Devices
Cell phone	There are two requirements of cell batteries, high specific energy and high specific power	Modern mobiles mostly use lithium ion batteries due to their specific power and energy
Laptops	Long cycle and shelf life, low discharging, withstand high temperature, rapid charging, low maintenance, should be sealed	Nickel–cadmium, nickel metal hydride and lithium-ion batteries
Digital SLR (single lens reflex) Cameras	They need lighter batteries with more power	Lithium-ion batteries are used because they can hold power more than 40% and these are very light
Toys, remotes, the game controllers and all gadgets	Toys need a lot of energy High-performance batteries are required, such as lithium and alkaline batteries.	Lithium-ion batteries are used due to their highest performance
WSNs sensors	Peak currents are needed during signal transmission and reception. WSNs need a low cost, small size, and portability.	lithium-ion batteries and supercapacitors.
Low cost microelectronics	Energy density, specific energy and cyclic life should be high.	Micro lithium-ion batteries, supercapacitors.
Low cost micro-energy harvesting	Energy density, specific energy and cyclic life should be high.	Lithium-ion battery and supercapacitors

## Data Availability

Not applicable.
